# Golgi ELMO1 binds QUA1, QUA2, GAUT9, and ELMO4 and is required for pectin accumulation in Arabidopsis

**DOI:** 10.1371/journal.pone.0293961

**Published:** 2023-11-08

**Authors:** Bruce D. Kohorn, Nuoya Yang, Margaret Weinstock, Garrison Asper, Isabel Ball, Devaki Rajiv

**Affiliations:** Department of Biology, Bowdoin College, Brunswick, ME, United States of America; VIT University, INDIA

## Abstract

Pectin and its modification influence the plasticity and strength of the plant cell wall controlling cell adhesion, size, shape, and pathogen resistance. The Golgi membrane anchored QUA1, QUA2, and GAUT9 Golgi enzymes synthesize and esterify pectin, which is then secreted and selectively de-esterified to potentiate structure influencing crosslinks in the cell wall. Mutations in members of the family of non-enzymatic ELMO Golgi membrane proteins lead to a reduction of pectin levels, cell adhesion, and hypocotyl tensile strength. Results from immunoprecipitation of Golgi protein complexes reveal that ELMO1-GFP is associated with pectin biosynthesis and modifying enzymes QUA1, QUA2, and GAUT9. In a yeast two and three hybrid assay, ELMO1 can bind directly to QUA1, GAUT9 or ELMO4, but QUA1, QUA2 or GAUT9 do not bind to each other. A yeast 3 hybrid assay provides evidence that ELMO1 can mediate the binding of QUA1 and QUA2. Taken together, these results indicate that the 20 kDa ELMO1 serves to facilitate some aspect of pectin synthesis and modification that leads to sufficient accumulation to allow cell adhesion, and we speculate that ELMOs help to scaffold key enzymes in this process.

## Introduction

The extra cellular matrix or cell wall of plants is composed of a complex interconnected array of pectin, cellulose, hemicellulose, and protein that influence the structure and flexibility of the wall, and defense against pathogens [1–4]. Pectins are comprised of a family of galacturonic acid polymers that differ in their repeating units and modifications, and are synthesized in the Golgi by a large family of Galacturonosyltransferase (GAUTs) and modifying enzymes including the QUASIMODO (QUA, methyltransferases) family [5]. Pectins are secreted and then selectively de-esterified to produce a highly regulated crosslinking that changes the properties of the wall through development and environmental challenges [6–9]. GAUT1 and GAUT7 are thought to form a complex [10] to potentiate activity [11] in the Golgi, but while numerous other enzymes are assumed to sequentially assemble pectin, it has not been clear how their activities are coordinated, and it is possible that scaffold proteins are involved. Scaffolds work to anchor proteins in a complex so that they can be properly linked to coordinate their activities, and are involved in executing signal transduction and enzymatic pathways in efficient and specific processes [12]. In the yeast *S*. *cerevisiae*, the scaffold protein Ste5 forms a multimolecular complex organizing a MAPK cascade [13,14]. c-Jun NH2-terminal kinase (JNK) associated protein and protein Kinase Suppressor of Ras 1 (KRS1) act as MAPK cascade scaffolds in mammalian cells [15,16] and Receptor for Activated C Kinase 1 (RACK1) is a scaffold attached to the plasma membrane and facilitates a kinase cascade involved in plant immunity [16]. Metabolons, or clusters of ordered enzymes are thought to increase efficiency and substrate specificity in a number of metabolic pathways and these too are organized by scaffold proteins [17]. Scaffold proteins may be involved in the synthesis of numerous secondary compounds in plants and enzymatic complexes synthesizing flavonoids in Arabidopsis suggest the potential for scaffolds to act as organizers to effect function [17,18]. Arabidopsis membrane steroid-binding proteins (MSBPs) may act as a scaffold that associate themselves as heteromers on the ER membrane and organize all three P450 enzymes, C4H, C3H, and F5H into a cluster that synthesize lignin, an important component of cell walls [19]. We report here on a Golgi protein ELMO1 that binds to multiple Golgi proteins that synthesize and modify Homogalacturonan and hence has the potential to serve as a scaffold for these enzymes. Loss of function of ELMOs leads to less pectin accumulation, and reduced cell adhesion.

## Materials and methods

*Mutant Strains* are all in an *Arabidopsis thaliana col*. background, and are described in [20]. *elmo1 (*At2g32580) has a point mutation in the 3’ splice junction of intron 2 and was isolated through a screen for adhesion mutants [20]. *elmo2*,*3*,*4* and *5* have T-DNA insertions in their transcribed regions as described [20] and were obtained from the Arabidopsis Stock Center. The following lists the gene name, gene number and T-DNA line; *ELMO2*, At1g05070, Salk205719C; *ELMO3*, At4g04360, Salk20486C; *ELMO4*, At4g30996, Salk 1398222; *ELMO5*, At2g24290, Salk 084405.

### Determination of pectin methylation and acetylation

Pectin levels were determined by extracting from Biological triplicates of 5 day old dark grown hypocotyls an Alcohol Insoluble Fraction (AIR) and determining total uronic acid content as described [20]. Briefly, dark grown seedlings were immersed in 96% ethanol and incubated at 80°C for 15 min, homogenized using a ball homogenizer for 20 min, centrifuged for 15 min at 20,000xg, and the supernatant was removed and the pellet was re-suspended in acetone and centrifuged for 15 min at 20,000xg. The supernatant was then removed and the pellet was re-suspended in methanol:chloroform (2v:3v) and shaken overnight. Samples were then centrifuged for 15 min at 20,000xg and the supernatant was removed. The pellet was then re-suspended sequentially in 100%, 65%, 80%, and 100% ethanol. After each re-suspension samples were centrifuged at 20,000xg for 15 min and the supernatant was removed and the pellet of AIR (Alcohol Insoluble Residue) dried under vacuum. This AIR was saponified overnight in 200μl of 0.05 M NaOH and the samples were centrifuged at 4°C, 10,000g, 10 min. The pellet was then washed twice with 70% ethanol (to remove residual NaOH) and twice with acetone at room temperature. The residual pellet was air dried under vacuum and extracted with Ammonium Oxalate as described [20]. Ammonium oxalate extracted uronic acid content was determined according to [20].

For esterification and acetylation measurements, 500 μl of 50 mM NaOH in H_2_O was added to 5 mg of AIR and allowed to incubate overnight at 4° C. The samples were centrifuged for 15 minutes at 14,000 rpm, and 400 μl of supernatant was removed for analysis. 100 μl of D_2_O and 100 μl of internal standard [1 mg/ml Trimethylsilypropanoic acid, TPS, in H_2_O) was added to each sample. The resulting 600 μl samples were added to Norell NMR tubes. All ^1^H NMR spectra were obtained using a Bruker AVANCE 400 MHz spectrometer with a spectral width of 8278.15 Hz and 32K points to give an acquisition time of 1.98 seconds. 64 scans were completed. Each spectra was accompanied with WATERGATE solvent suppression with a T_1_ delay of 4 seconds to ensure full proton relaxation [21]. The collected FID was Fourier transformed and processed with Bruker TopSpin 1.3 software with 0.30 Hz line broadening. Each spectra was phased and baseline corrected with a 5 degree polynomial. The TSP peak was set to 0 ppm, and methanol and acetic acid peaks were determined to be at 1.922 ppm and 3.365 ppm consistent with previous literature [22]. The peak area was determined using manual integration, with the TSP peak assigned an area of 1.0 for each spectra. The area of a peak is dependent on both the amount of analyte in solution, as well as the number of hydrogens contributing to each particular peak. The following formula was used to obtain the mass of both methanol and acetic acid released from AIR. A_*x*_× (EW_*x*_ / mg_*x*_) = A_TSP_ × (EW_TSP_ / mg_TSP_). A_*x*_ is the peak area of the analyte of interest. A_TSP_ is the peak area of the internal standard, and was always equal to 1.0. EW_*x*_ is the equivalent weight of the analyte while EW_TSP_ is the equivalent weight of TSP. Equivalent weight is defined as the molecular weight divided by the number of hydrogens contributing to the peak [22,23].

*Propidium iodide and Ruthenium Red staining* were as described [20]. Five-day old dark grown seedlings were stained with 10 μg/ml propidium iodide for 15 min, and then removed to 5 ml of dH_2_0. Hypocotyls were imaged under a Leica SP8 confocal microscope using a 10x objective, and 514 nm excitation and 617 nm emission spectra. Leica SP8 software generated a Z stack. Five-day old dark grown were also stained with Ruthenium Red dye (Sigma Corp., 0.5 mg/ml in dH_2_O) for 2 min. in a 10 ml microtiter growth plate, washed twice with 5 mls of dH_2_O, and observed under a dissecting microscope.

### Tensiometer measurements

A motorized Siskiyou translation stage (DR1000 single-axis digital readout, a 7600-X translation stage and a MC1000e-1 controller) which gives the capability of a 20 mm movement with +/-2 micrometer positioning reliability and +/-5 micrometer backlash. Force was measured with a Kyowa Ultra Small-capacity Load Cell LTS-50GA with a range of +/-50 g and a repeatability of +/- 0.25 g, and a rate of 0.16 mm/sec. Each end of a hypocotyl was superglued to an acetate sheet clamped to each force transducer. The signals from the force transducers were recorded with Spike2 version 7.03 software. At least 10 biological replicates were measured for each genotype.

### Immunoprecipitation

Arabidopsis seeds were incubated for 2 day 4°C, then 4 hr light at 21°C, and then for 5 days dark at 21°C in liquid MS 1% sucrose. The hypocotyls were frozen in liquid nitrogen and then ground in a mortar and pestle. The powder was incubated with agitation at 4°C for 10 min in 50 mM Hepes pH 7.5, 1 mM EDT, 2 mM EGTA, 1 tablet of Roche Inhibitor cocktail (per 10 ml). The solution was centrifuged at 10,000 xg for 10 min, and the supernatant centrifuged at 100,000 xg for 60 min at 4°C. The pellet (microsomes) was resuspended in 10 mM Tris-HCl pH 7.5, 150 mM NaCl, 1 mM EDTA, 0.5% NP40 and incubated with Magnetic agarose coupled Llama anti-GFP antibody (BullDog Bio Corp.) for 1 hr. at 4°C. The beads were washed 6 times in the microsome resuspension buffer and the pellet was subjected to LC-MS/MS.

*SWATH quantitative LC-MS/MS* was as described [24] and Arabidopsis proteins identified by searching the TAIR10 data base.

### Yeast 2 and 3 hybrid assay

ELMO1, ELMO4, QUA1, QUA2 and GAUT9 were PCR amplified and ligated to either pLEXA (XhoI site) or pACT (EcoRI site) by Gibson assembly [25]. *S*.*cerevisiae* L40 (*MATa his3Δ200 trp1-901 leu2-3112 ade2 LYS*::*(4lexAop-HIS3) URA3*::*(8lexAop-LacZ)GAL4*) was transformed [26] sequentially with the appropriate combination of plasmids and grown in selective media and 2% glucose until log phase. Cells were counted with a hemocytometer, washed 3x in PBS (0.14 *M* NaCl, 0.2 m*M* KCl, 10 m*M* Na_2_HPO_4_, 1.8 m*M* KH_2_PO_4_, pH 7.4) and diluted to the same concentration (2.5 x 10^8^ cells/ml) in PBS, and then 10 ul of 5 serial 10 fold dilutions were plated on the indicated media. For the yeast 3 hybrid assay, ELMO1 was cloned by Gibson assembly using the following primers first into the HindIII/XbaI site of pYES NTA, and then the GAL1 promoter and ELMO1 were PCR amplified and ligated to the AATII site of pACT already harboring the indicated fusion protein, using Gibson assembly. Cells were grown overnight in selective media and 2% galactose, serially diluted as above, and then plated on the indicated media. 10 mM 3AT was included as indicated.

### Protein localization

A Gaut9-GFP fusion was created by cloning the GAUT9 coding region using Gibson assembly into pCambia 1302. GOT1-RFP [27] was obtained from the Arabidopsis stock center. Both plasmids were co-transformed into protoplasts isolated from 2 week old soil grown Arabidopsis leaves, as described [28]. 10 ml enzyme solution (0.4M Manitol, 20 mM KCL, 20 mM MES pH 5.7) containing 100 mg of cellulase and 20 mg of macerozyme were heated at 55°C for 10 minutes, 30 μl of 3M CaCl_2_ and 10 mg BSA (fraction V Sigma Chem Corp.) were then added, and the solution was filter sterilized. Ten, two-week-old leaves were placed in enzyme solution and cut into fine strips and then placed into a 10 ml well plate with 5 ml of sterilized enzyme solution containing the cellulase and macerozyme. The sample was placed on an orbital shaker at 40 rpm, and a vacuum was applied to the leaves and solution for 5 minutes. The vacuum was then released, and the well plate was left on the orbital shaker for 1.5 hours. Finally, the shaker was increased to 80 rpm for 1 minute. A blunted 1 ml pipette tip was used to gently remove the leaf solution and pipette it through a 200 μm nylon mesh into a 50 ml conical tube. The tube was centrifuged at 100xg for 2 minutes, followed by careful supernatant removal and cell resuspension with 5 ml cold W5 (150 mM NaCl, 120 mM CaCl_2_, 5 mM KCl, 2 mM MES pH 5.7). Protoplasts were centrifuged again at 100xg for 2 minutes, the supernatant was removed gently, and the cells were resuspended carefully in 5 mls cold W5. The protoplast solution was left on ice for 30 minutes and centrifuged at 100xg for 2 minutes, the supernatant was removed, and the protoplasts were resuspended in 500 μl MMG (0.4M Mannitol, 15 mM MgCl_2_, 4 mM MES pH 5.7). To a 1.5 ml tube containing 10 μg of each DNA in 10 μl water, 100 μl of protoplasts and 110 μl of PEG solution (40% PEG4000, 200 mM Mannitol, 10 mM CaCl_2_) were added and gently mixed, followed by a 30-minute incubation at room temperature. Samples were then diluted with 440 μl room temperature W5 and carefully mixed, centrifuged at 100xg for 3 minutes, the supernatant was carefully removed, and the protoplasts were resuspended in 1 ml W5, and incubated at 25 C°under room light overnight to produce new GFP and RFP tagged proteins prior to confocal imaging.

### Confocal microscopy

Protoplasts visualized on a Leica SP8 confocal microscope using a 40x objective and processed using the Leica SP8 software. GFP was visualized with 488 nm excitation and 515 nm emission, and RFP with 550 nm excitation and 700 nm emission using sequential scanning. At least 6 biological replicates were measured for each genotype. The JACoP plugin of Image J [29] was used to determine colocalization between the GFP and RFP channels. The same threshold value was chosen for both channels and the Manders overlap coefficient M1 was recorded. The M1 value indicates the fraction of GFP overlap with RFP [30], and was calculated for a minimum of six replicates for each cell type. GraphPad Prism was used to average the replicates of each cell type, and a one-way ANOVA followed by a Dunnett’s Test was performed in GraphPad Prism to compare all cell types to WT.

### RNA extraction and RNA Seq

RNA was extracted from *elmo1*^*-/-*^, *elmo2*^*-/-*^, *elmo3*^*-/-*^, *elmo4*^*-/-*^, *elmo5*^*-/-*^, *elmo1*^*-/-*^*2*^*-/-*^, *elmo1*^*-/-*^*3*^*-/-*^, *elmo1*^*-/-*^*5* ^*-/-*^, and *elmo4*^*-/-*^*5* ^*-/-*^ mutants (biological triplicates) using the RNeasy Plant Mini Kit according to provided instructions (Qiagen Corp). RNA seq was performed by Novagene Corp.

## Results and discussion

### *elmo* mutants have reduced levels of pectin

**Fig 1:**
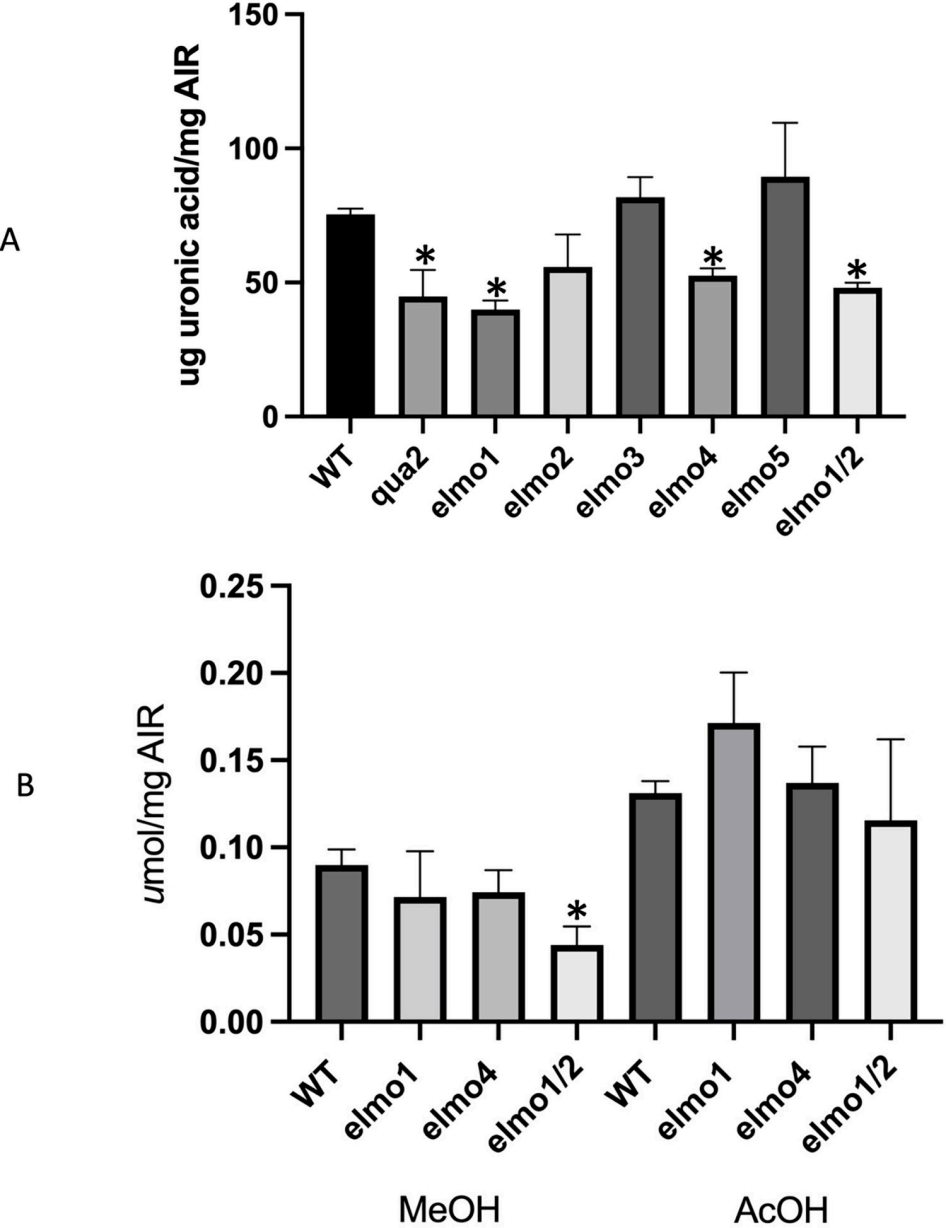
A) Pectin was measured by extracting total uronic acid from an alcohol insoluble fraction (AIR) from the indicated genotype (ANOVA F(3,8) = 8.722 p = 0.0067). B) The level of esterification or acetylation was determined by the NaOH release of methanol or acetic acid from AIR of the indicated genotype (ANOVA F93,8) = 1.901 p = 0.2079). Asterisks indicate a difference with WT, 3 biological replicates were measured per genotype. Bars indicate standard deviation.

Loss of function *qua1*, *qua2*, and *elmo1* alleles in Arabidopsis lead to reduced cell adhesion in the hypocotyl, and both *qua1* ^*-/-*^ and *qua2* ^*-/-*^ have a reduced level of pectin [20,31–33]. *elmo1* ^*-/-*^ appeared to only have reduced mannose in a whole cell wall analysis, however growth conditions varying between labs can affect pectin measurements [33,34] and the pectin content was reevaluated for *qua2* ^*-/-*^ and *elmo1* ^*-/-*^. Pectin was indeed reduced in both *qua2* ^*-/-*^ and *elmo1* ^*-/-*^ hypocotyls since the total uronic acid levels per gram of cell wall (Alcohol Insoluble Residue, AIR) was reduced relative to WT (Fig 1A). Reduced pectin was seen only in mutants that showed a visible adhesion phenotype [20], including *elmo1*^*-/-*^, *elmo2*^*-/-*^, *elmo4*^*-/-*^ and the double mutant *elmo1*^*-/-*^ *elmo 2*^*-/-*^ (Fig 1A). Further analysis also indicated that *elmo1*^*-/-*^ *elmo 2*^*-/-*^ but not *elmo1* ^*-/-*^ or *elmo4* ^*-/-*^ have reduced levels of methyl esterification but WT levels of acetylation per ug of AIR (Fig 1B). Esterification can also have effects on crosslinking and hence cell wall properties and cell adhesion [9,35,36], but it is unclear if the assay is sufficiently sensitive to identify a change in single mutants, or if a more severe allele or a double mutant is needed. Nevertheless, reduced levels of pectin in *elmo* mutants correlate with a loss of cell adhesion.

### Double *elmo* mutants affect cell adhesion

**Fig 2:**
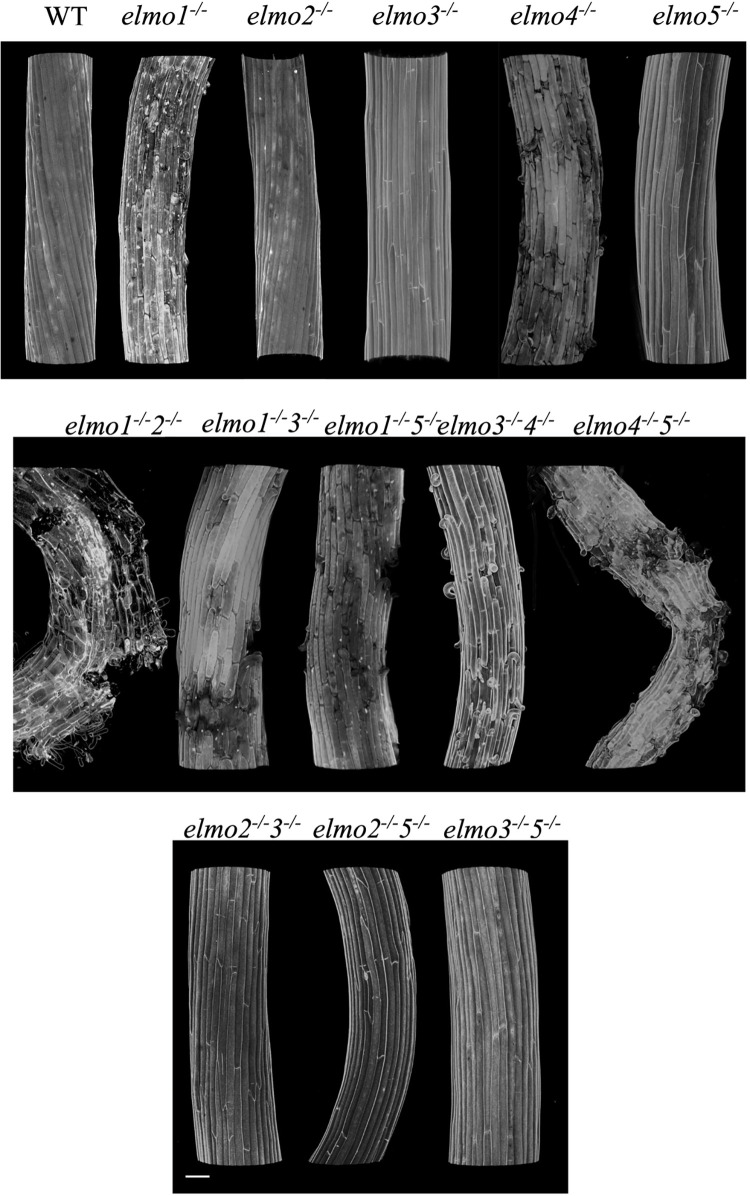
Double *elmo* mutants have stronger adhesion defects. Three dimensional reconstruction by confocal microscopy of propidium iodide stained hypocotyls of the indicated genotype. Bar indicates 50 mm, images were taken independently and combined.

Given the high level of conservation within the ELMO family, that some single mutants lack phenotype, and that *elmo1*^*-/-*^ *elmo 2*^*-/-*^ is more severe than *elmo1*^*-/-*^, additional double mutants were generated to determine which pairs might provide redundancy. Ruthenium Red penetrates mutant but not WT hypocotyls to bind to pectin and is used as an indicator of a loss of cell adhesion [20,37,38]. S1 Fig in S1 File shows Ruthenium Red stained dark grown hypocotyls imaged under a dissecting microscope, and Fig 2 shows confocal images of propidium iodide stained dark grown hypocotyls of pairwise *elmo* mutants. The single mutants *elmo1*^*-/-*^ *and elmo4*^*-/-*^ have noticeable cell adhesion defects exhibiting curling and detached cells, while *elmo2*^*-/-*^, *elmo3*^*-/-*^ *and elmo5*^*-/-*^ have no phenotype [20]. The double mutants *elmo1*^*-/-*^ *elmo 2*^*-/-*^, *elmo1*^*-/-*^ *elmo 3*^*-/-*^, *elmo1*^*-/-*^ *elmo 5*^*-/-*^, and *elmo4*^*-/-*^ *elmo 5*^*-/-*^ have more pronounced effects than either of the single mutants. Combined with the varying tissue specific expression of the family members [20], it appears there is functional redundancy within the family and each isoform may provide similar functions. The adhesion phenotype is also accompanied by a reduction in the hypocotyl length in mutants that exhibit a visible phenotype, and these measurements are shown in S2 Fig in S1 File. Root length is only affected in the double mutants *elmo1*^*-/-*^ *elmo 2*^*-/-*^ and *elmo4*^*-/-*^ *elmo 5*^*-/-*^ *(*S2 Fig in S1 File*)*. RNA seq was performed on hypocotyl RNA from *elmo1*^-/-^, elmo4^-/-^ and *elmo1*^*-/-*^ *elmo 2*^*-/-*^ to determine if there were increases in gene expression from *ELMO* family members to compensate for a reduction in one or two isoforms (S3 Fig in S1 File). While the analysis confirmed a reduction in *ELMO* expression in the corresponding mutant, no compensatory changes were detected, nor were there any changes in genes encoding the major biosynthetic enzymes for pectin. All *elmo* mutants revealed numerous increases or decreases in gene expression that did not provide a recognizable pattern using either visual inspection or GO analysis (S3 Fig in S1 File).

### Tensile strength of *elmo* hypocotyls

**Fig 3:**
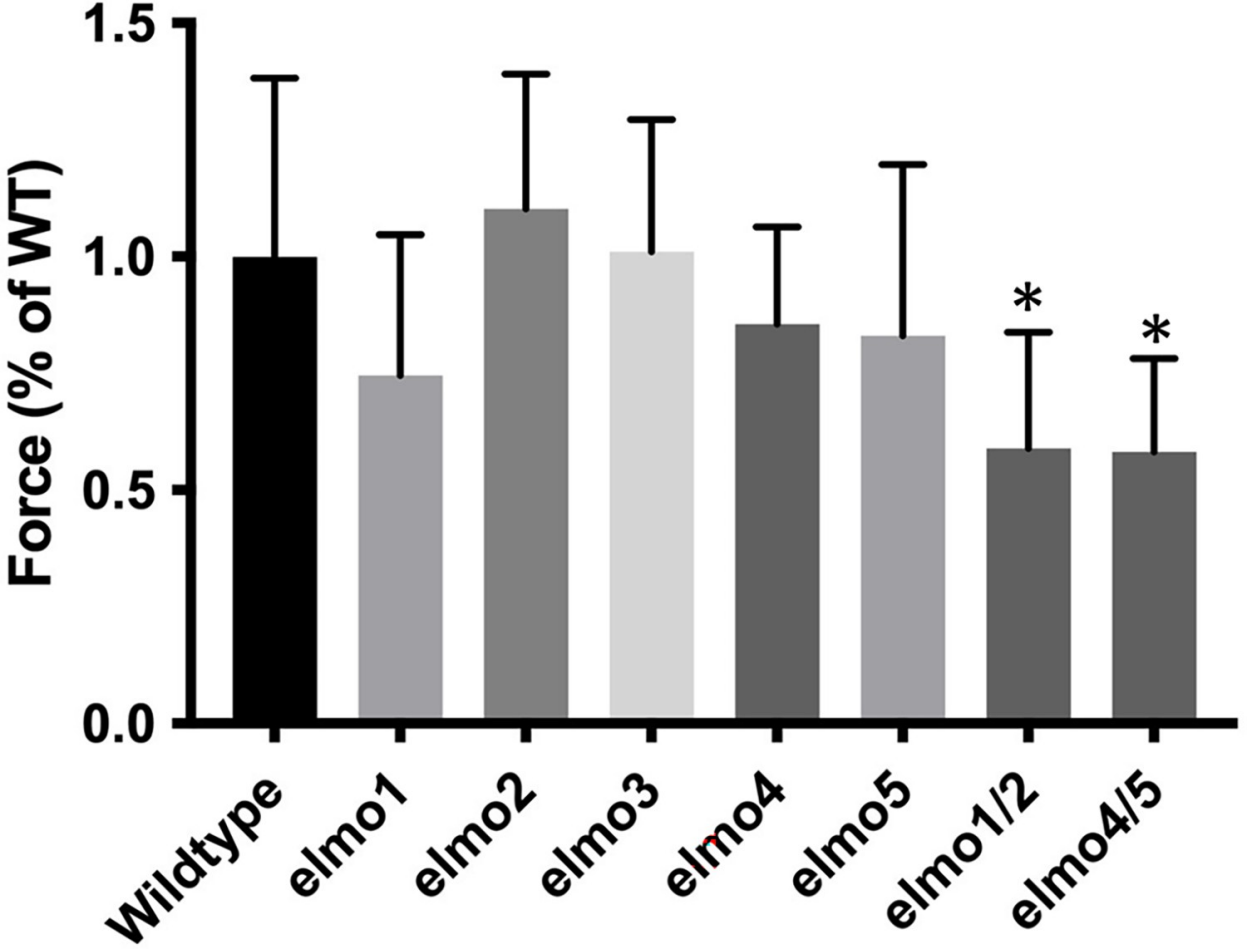
*elmo* mutations weaken hypocotyl strength. Hypocotyls were stretched with a Tensiometer, and the breaking point was expressed as percentage of wild type (WT). Asterisks indicate a difference with WT. A minimum of 10 biological replicates per genotype were measured. (ANOVA, F(12,227) = 4.354 p<0.0001). Bars indicate standard deviation.

Given a reduced cell adhesion in *elmo* mutants it was predicted that the tensile strength of the hypocotyl would be compromised in mutants with a visible phenotype. Hypocotyls of the indicated phenotype (Fig 3) were stretched using a tensiometer, and the pulling force required to break a hypocotyl was recorded as a percentage of wild type. Only the double mutants *elmo1*^*-/-*^ *elmo* 2^-/-^ and elmo4^-/-^ *elmo* 5^-/-^ showed a significant difference to wild type, indicating that loss of adhesion leads to a compromised structure and tensile strength of hypocotyl cell walls. It is likely that the assay was insufficiently sensitive to distinguish the strengths of single mutants from wild type.

### ELMO1 is associated with multiple pectin related enzymes in the Golgi

ELMO1 is predicted to be 20 kDa in molecular weight, with two helical domains and no enzyme activity [20], and hence may well serve to bind to other proteins in the Golgi. To explore how ELMO1 is involved in the accumulation of pectin, we next determined what proteins ELMO1 associates with. An ELMO1-GFP was expressed and complemented an *elmo1* ^*-/-*^ mutant [20], and hypocotyl microsomal extracts from these transformed plants were used for immunoprecipitation with anti-GFP antiserum, and the isolated proteins were sequenced using SWATH quantitative LC-MS/MS [24] and identified in the Arabidopsis proteome. Hypocotyls expressing only GFP were used for comparison, and S4 Fig in S1 File shows the results. Peptides representing five proteins were immunoprecipitated far more in the ELMO-GFP than the GFP samples (fold change shown after each protein) and with a p < 0.01; ELMO4 (60 fold), ELMO1 (40 fold), QUA2 (15 fold), QUA1 (or GAUT8 11 fold) and GAUT9 (7 fold). There were only two other proteins having a p< 0.01 and a greater than 2 fold enrichment; histone deacetylase and HSP70. Histone deacetylase is a nuclear protein and is likely to represent background non-specific binding, and the significance of ER hsp70 binding is not known.

### ELMO1 is not a retention protein

**Fig 4:**
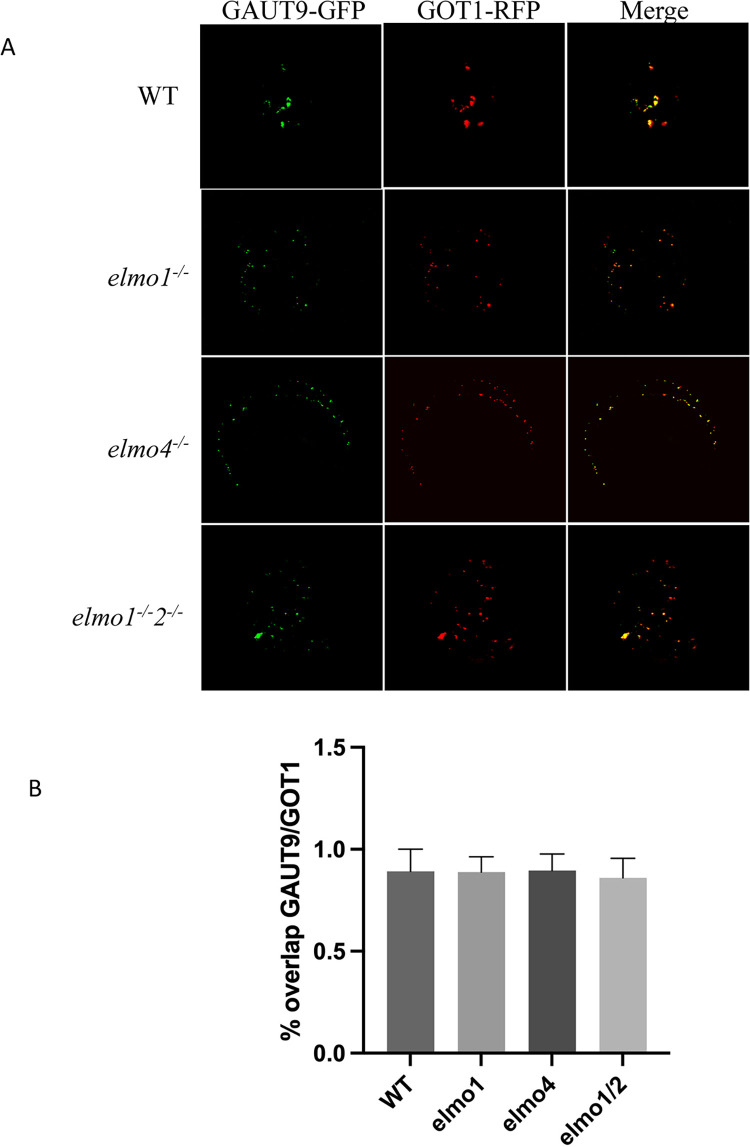
ELMO1 is not a Golgi retention protein. A) ELMO1 associated GAUT9-GFP was expressed in the indicated genotype with a Golgi marker GOT1-RFP and detected by confocal microscopy. B) A colocalization index (M1) was measured from images in A, and no mutants were different from WT. A minimum of six replicates for each cell type (ANOVA F(3,27) = 0.2558 p = 0.8565). Bars indicate standard deviation. Images were captured independently and arranged.

The function of ELMO1 binding to QUA1, QUA2 and GAUT9 could be to affect or organize enzyme activity and hence its absence leads to reduced levels of pectin and cell adhesion. Alternatively ELMO1 may be needed to retain these enzymes in the Golgi for them to provide sufficient pectin content. To determine if ELMO1 serves as a retention protein, a GAUT9-GFP fusion was expressed in an *elmo1*^*-/-*^, *elmo4*^*-/-*^, *elmo1*^*/-*^ *elmo*2^-/-^ or WT plant cell, and the location of GAUT9-GFP was identified by confocal microscopy. Cells were also transformed with a GOT1-RFP Golgi membrane marker [27], and a colocalization index (M1) [29] for the GOT1 and GAUT9 was measured. The results in Fig 4 show that there was no difference in GAUT9 localization in *elmo1*^*-/-*^, *elmo4*^*-/-*^, or *elmo1*^-/-^ *elmo*—2^-/-^ mutants, relative to WT, indicating that GAUT9 is retained in the Golgi in the absence or a reduction of ELMO proteins. Thus ELMO1 likely plays a role distinct from retention of the proteins to which it is bound.

### ELMO1 binds QUA1, GAUT9 and ELMO4 in yeast 2 hybrid assay

**Fig 5:**
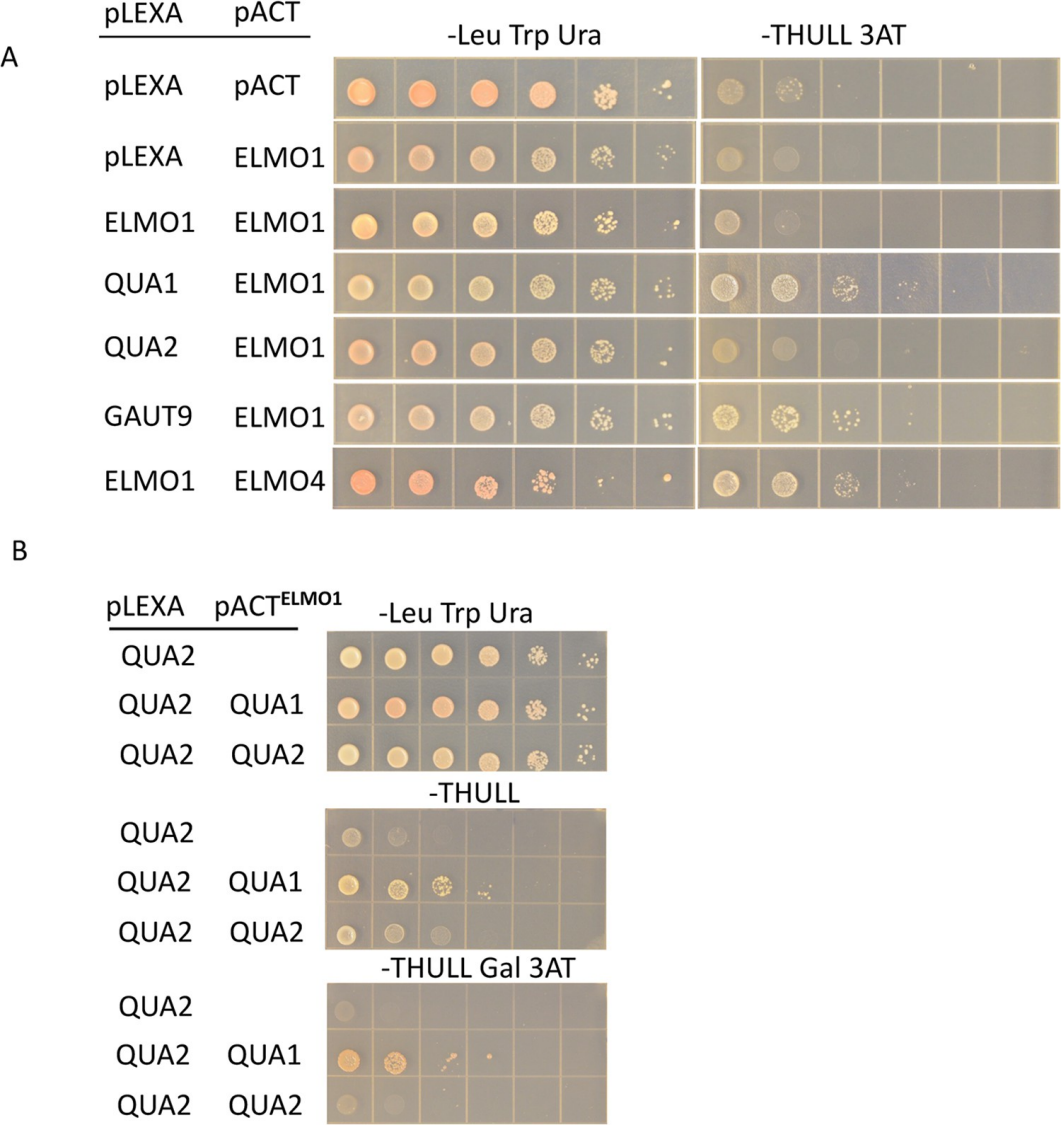
Yeast 2 and 3 hybrid assays shows ELMO1 interacts with QUA1, QUA2, GAUT9 and ELMO4. Cells were grown to the same density and serial dilutions were spotted on media. A) Yeast 2 hybrid assay with the indicated fusion proteins (left column) on either media selecting for plasmids (-Leu Trp, Ura) or media also requiring expression of HIS (-THULL 3AT). B) Yeast 3 hybrid assay where ELMO1 is expressed with the GAL1 promoter with the combinations shown on the left columns.

The immunoprecipitation assay does not distinguish if ELMO1 is bound individually with each of the pectin biosynthetic enzymes, or if ELMO1 is bound to one or a few proteins that then form a larger complex containing QUA1, QUA2, GAUT9 and ELMO4. Bimolecular fluorescence in plant cells could be used to ask what proteins ELMO is proximal to, but would not exclude the possible involvement of other plant proteins in the binding. The yeast two hybrid assay [39,40] was therefore used to test a direct interaction between ELMO1, ELMO4, QUA1, QUA2 and GAUT9. Each protein was expressed in yeast as a fusion to either the lexA DNA binding domain (pLexA), or the GAL4 activation domain (pACT) to effect the activation of the *HIS* gene, and assayed on media lacking histidine (his do), or his do and 3-Aminotriazole (3AT) to provide a more stringent assay. In each fusion, the amino-terminal membrane anchor of the Golgi proteins was deleted to allow for effective nuclear localization. Cells were grown to log phase in the presence of histidine, diluted to the same concentration, and then serial dilutions were spotted on media either not requiring interaction (lacking ura leu trp), or media also lacking histidine with or without 3AT (THULL). All ELMO4-plexA constructs activated without a pACT partner and were therefore not useful (S5 Fig in S1 File). The results shown in Fig 5A and S5 Fig in S1 File indicate that ELMO1 can bind directly to QUA1, GAUT9 or ELMO4, but QUA1, QUA2 or GAUT9 do not bind to each other. However, a lack of interaction in the yeast 2 hybrid assay cannot be taken as a true lack of interaction in planta. To determine if ELMO1 might cause QUA1, QUA2 or GAUT9 to bind to each other, a yeast 3 hybrid assay was developed where a GAL1 promoter driving ELMO1 was added to the pACT plasmid also expressing either QUA1, QUA2 or GAUT9. The results (Fig 5B and S5 Fig in S1 File) show that a QUA1, QUA2, ELMO1 combination can activate HIS expression, indicating that ELMO1 can cause QUA1 and QUA2 to bind to each other, where in the absence of ELMO1 there is no QUA1 and QUA2 interaction. Other combinations of enzymes expressed in yeast were not sufficient to activate HIS expression and provide no evidence of interaction.

Taken together, the immunoprecipitation, yeast two and three hybrid results raise several possible arrangements that ELMO1 might hold. It is possible that ELMO1 is bound to individual molecules of GAUT9 and ELMO4, and separately serves to bind QUA1 and QUA2 together. Alternatively, ELMO1 may sit at the interface of individual components of a complex that contains GAUT9, QUA1, QUA2, and as such could serve as a scaffold. ELMO1 binds to ELMO4 of similar structure and sequence, and this might indicate that multiple ELMO1 and 4 molecules may make up the interface of such a complex. Indeed since ELMO family members are only 20 kDa, it might require multiple molecules to cause the binding of the far larger QUAs and GAUTs. However, the results at present can not distinguish these and perhaps other more complex models of an ELMO protein arrangement. The stoichiometry and structure of any ELMO complexes awaits the analysis of crystals, and the ability of ELMO1 and ELMO4 to form a lattice to scaffold the enzymes is possible but remains to be determined.

QUA1 is a galacturonosyltransferase responsible for homogalacturonic acid pectin synthesis [32], while QUA2 is a methyltransferase [33], and the results here indicate that the two activities are linked. If ELMOs do provide a scaffold for a larger biosynthetic or modifying complex, it follows that GAUT9, also a galacturonosyltransferase, is sequential to QUA1 and 2, but an order of action cannot be determined from the results reported here. While the combined glycosyltransferase and methyltransferase families includes over 100 isoforms, it is striking that ELMO appears to only associate with QUA1, QUA2 and GAUT9. This work raises the possibility that there are additional binding proteins that might serve other members of the GAUT and QUA families.

Since mutants in ELMO reduce the levels of pectin, esterification, and adhesion, it is predicted that the association of ELMO with GAUT9, QUA1, or QUA2 affects their activity. However, while ELMO1 co-immunoprecipitates with ELMO4, QUA1, QUA2 and GAUT9, the existence of in-planta interactions requires further verification, and the nature and mechanism by which this binding might occur remains to be determined.

## Supporting information

S1 File **SI Fig 1**. Ruthenium Red stained hypocotyls for the indicated genotype. Scale bar indicates 1 mm. **SI Fig 2**. *elmo* mutants have reduced hypocotyl root lengths. 5 day dark grown hypocotyls were imaged using a dissecting microscope and the hypocotyl and roots were measured. Asterisks indicates significant difference from WT (ANOVA hypocotyl; F (12,337) = 91.89 p<0.0001. root; F(12, 312) = 20.78 p<0.0001). ß **SI Fig 3 and Fig 4**. **Zip file containing RNA seq and immunoprecipitation data files.** SI Fig 3; Excel files of RNA seq of RNA from *elmo1*^*-/-*^, *elmo4*^*-/-*^, *elmo1*^*-/-*^ *elmo4*^*-/-*^ and WT dark grown hypocotyls. SI Fig 4; Excel files of ELMO1-GFP immunoprecipitated from dark grown hypocotyls, and proteins sequenced using SWATH quantitative LC-MS/MS. **SI Fig 5**. Yeast two hybrid assay for the indicated combinations of expressed proteins.
(PDF)

S2 File (ZIP)

## References

[1] Cosgrove DJ. Building an extensible cell wall. Plant Physiol. 2022;189(3):1246–77. doi: 10.1093/plphys/kiac184 35460252 PMC9237729

[2] Cosgrove D. Plant biology: Peering deeply into the structure of the onion epidermal cell wall. Curr Biol. 2022;32(11):R515–R7. 35671723 10.1016/j.cub.2022.04.087

[3] Engelsdorf T, Gigli-Bisceglia N, Veerabagu M, McKenna JF, Vaahtera L, Augstein F, et al. The plant cell wall integrity maintenance and immune signaling systems cooperate to control stress responses in Arabidopsis thaliana. Sci Signal. 2018;11(536). doi: 10.1126/scisignal.aao3070 29945884

[4] Anderson CT. We be jammin’: an update on pectin biosynthesis, trafficking and dynamics. J Exp Bot. 2016;67(2):495–502. doi: 10.1093/jxb/erv501 26590862

[5] Amos RA, Mohnen D. Critical Review of Plant Cell Wall Matrix Polysaccharide Glycosyltransferase Activities Verified by Heterologous Protein Expression. Frontiers in plant science. 2019;10:915. doi: 10.3389/fpls.2019.00915 31379900 PMC6646851

[6] Huerta AI, Sancho-Andres G, Montesinos JC, Silva-Navas J, Bassard S, Pau-Roblot C, et al. The WAK-like protein RFO1 acts as a sensor of the pectin methylation status in Arabidopsis cell walls to modulate root growth and defense. Molecular plant. 2023;16(5):865–81. doi: 10.1016/j.molp.2023.03.015 37002606 PMC10168605

[7] Zhang X, Guo H, Xiao C, Yan Z, Ning N, Chen G, et al. PECTIN METHYLESTERASE INHIBITOR18 functions in stomatal dynamics and stomatal dimension. Plant Physiol. 2023;192(2):1603–20. doi: 10.1093/plphys/kiad145 36879425 PMC10231589

[8] Coculo D, Del Corpo D, Martinez MO, Vera P, Piro G, De Caroli M, et al. Arabidopsis subtilases promote defense-related pectin methylesterase activity and robust immune responses to botrytis infection. Plant Physiol Biochem. 2023;201:107865. doi: 10.1016/j.plaphy.2023.107865 37467533

[9] Coculo D, Lionetti V. The Plant Invertase/Pectin Methylesterase Inhibitor Superfamily. Frontiers in plant science. 2022;13:863892. doi: 10.3389/fpls.2022.863892 35401607 PMC8990755

[10] Atmodjo MA, Sakuragi Y, Zhu X, Burrell AJ, Mohanty SS, Atwood JA 3rd, et al. Galacturonosyltransferase (GAUT)1 and GAUT7 are the core of a plant cell wall pectin biosynthetic homogalacturonan:galacturonosyltransferase complex. Proc Natl Acad Sci U S A. 2011;108(50):20225–30. doi: 10.1073/pnas.1112816108 22135470 PMC3250160

[11] Amos RA, Pattathil S, Yang JY, Atmodjo MA, Urbanowicz BR, Moremen KW, et al. A two-phase model for the non-processive biosynthesis of homogalacturonan polysaccharides by the GAUT1:GAUT7 complex. J Biol Chem. 2018;293(49):19047–63. doi: 10.1074/jbc.RA118.004463 30327429 PMC6295712

[12] Good MC, Zalatan JG, Lim WA. Scaffold proteins: hubs for controlling the flow of cellular information. Science. 2011;332(6030):680–6. doi: 10.1126/science.1198701 21551057 PMC3117218

[13] Levchenko A, Bruck J, Sternberg PW. Scaffold proteins may biphasically affect the levels of mitogen-activated protein kinase signaling and reduce its threshold properties. Proc Natl Acad Sci U S A. 2000;97(11):5818–23. doi: 10.1073/pnas.97.11.5818 10823939 PMC18517

[14] Choi KY, Satterberg B, Lyons DM, Elion EA. Ste5 tethers multiple protein kinases in the MAP kinase cascade required for mating in S. cerevisiae. Cell. 1994;78(3):499–512. doi: 10.1016/0092-8674(94)90427-8 8062390

[15] Lee CM, Onesime D, Reddy CD, Dhanasekaran N, Reddy EP. JLP: A scaffolding protein that tethers JNK/p38MAPK signaling modules and transcription factors. Proc Natl Acad Sci U S A. 2002;99(22):14189–94. doi: 10.1073/pnas.232310199 12391307 PMC137859

[16] Su J, Xu J, Zhang S. RACK1, scaffolding a heterotrimeric G protein and a MAPK cascade. Trends Plant Sci. 2015;20(7):405–7. doi: 10.1016/j.tplants.2015.05.002 25986967

[17] Jorgensen K, Rasmussen AV, Morant M, Nielsen AH, Bjarnholt N, Zagrobelny M, et al. Metabolon formation and metabolic channeling in the biosynthesis of plant natural products. Curr Opin Plant Biol. 2005;8(3):280–91. doi: 10.1016/j.pbi.2005.03.014 15860425

[18] Burbulis IE, Winkel-Shirley B. Interactions among enzymes of the Arabidopsis flavonoid biosynthetic pathway. Proc Natl Acad Sci U S A. 1999;96(22):12929–34. doi: 10.1073/pnas.96.22.12929 10536025 PMC23169

[19] Gou M, Ran X, Martin DW, Liu CJ. The scaffold proteins of lignin biosynthetic cytochrome P450 enzymes. Nat Plants. 2018;4(5):299–310. doi: 10.1038/s41477-018-0142-9 29725099

[20] Kohorn BD, Zorensky FDH, Dexter-Meldrum J, Chabout S, Mouille G, Kohorn S. Mutation of an Arabidopsis Golgi membrane protein ELMO1 reduces cell adhesion. Development. 2021;148(10). doi: 10.1242/dev.199420 34015094 PMC8180255

[21] Piotto M, Saudek V, Sklenar V. Gradient-tailored excitation for single-quantum NMR spectroscopy of aqueous solutions. J Biomol NMR. 1992;2(6):661–5. doi: 10.1007/BF02192855 1490109

[22] Muller-Maatsch J, Caligiani A, Tedeschi T, Elst K, Sforza S. Simple and validated quantitative (1)H NMR method for the determination of methylation, acetylation, and feruloylation degree of pectin. J Agric Food Chem. 2014;62(37):9081–7. 25137229 10.1021/jf502679s

[23] Caligiani A, Acquotti D, Palla G, Bocchi V. Identification and quantification of the main organic components of vinegars by high resolution 1H NMR spectroscopy. Anal Chim Acta. 2007;585(1):110–9. doi: 10.1016/j.aca.2006.12.016 17386654

[24] Bond KH, Chiba T, Wynne KPH, Vary CPH, Sims-Lucas S, Coburn JM, et al. The Extracellular Matrix Environment of Clear Cell Renal Cell Carcinoma Determines Cancer Associated Fibroblast Growth. Cancers (Basel). 2021;13(23). doi: 10.3390/cancers13235873 34884982 PMC8657052

[25] Gibson DG, Young L, Chuang RY, Venter JC, Hutchison CA 3rd, Smith HO. Enzymatic assembly of DNA molecules up to several hundred kilobases. Nature methods. 2009;6(5):343–5. doi: 10.1038/nmeth.1318 19363495

[26] Gietz D, St Jean A, Woods RA, Schiestl RH. Improved method for high efficiency transformation of intact yeast cells. Nucleic Acids Res. 1992;20(6):1425. doi: 10.1093/nar/20.6.1425 1561104 PMC312198

[27] Geldner N, Denervaud-Tendon V, Hyman DL, Mayer U, Stierhof YD, Chory J. Rapid, combinatorial analysis of membrane compartments in intact plants with a multicolor marker set. Plant J. 2009;59(1):169–78. doi: 10.1111/j.1365-313X.2009.03851.x 19309456 PMC4854200

[28] Kohorn BD, Johansen S, Shishido A, Todorova T, Martinez R, Defeo E, et al. Pectin activation of MAP kinase and gene expression is WAK2 dependent. Plant J. 2009;60(6):974–82. doi: 10.1111/j.1365-313X.2009.04016.x 19737363 PMC3575133

[29] Bolte S, Cordelieres FP. A guided tour into subcellular colocalization analysis in light microscopy. J Microsc. 2006;224(Pt 3):213–32. doi: 10.1111/j.1365-2818.2006.01706.x 17210054

[30] Manders EM, Stap J, Brakenhoff GJ, van Driel R, Aten JA. Dynamics of three-dimensional replication patterns during the S-phase, analysed by double labelling of DNA and confocal microscopy. J Cell Sci. 1992;103 (Pt 3):857–62. doi: 10.1242/jcs.103.3.857 1478975

[31] Du J, Kirui A, Huang S, Wang L, Barnes WJ, Kiemle SN, et al. Mutations in the Pectin Methyltransferase QUASIMODO2 Influence Cellulose Biosynthesis and Wall Integrity in Arabidopsis. Plant Cell. 2020;32(11):3576–97. doi: 10.1105/tpc.20.00252 32883711 PMC7610292

[32] Bouton S, Leboeuf E, Mouille G, Leydecker MT, Talbotec J, Granier F, et al. QUASIMODO1 encodes a putative membrane-bound glycosyltransferase required for normal pectin synthesis and cell adhesion in Arabidopsis. Plant Cell. 2002;14(10):2577–90. doi: 10.1105/tpc.004259 12368506 PMC151237

[33] Mouille G, Ralet MC, Cavelier C, Eland C, Effroy D, Hematy K, et al. Homogalacturonan synthesis in Arabidopsis thaliana requires a Golgi-localized protein with a putative methyltransferase domain. Plant J. 2007;50(4):605–14. doi: 10.1111/j.1365-313X.2007.03086.x 17425712

[34] Krupkova E, Immerzeel P, Pauly M, Schmulling T. The TUMOROUS SHOOT DEVELOPMENT2 gene of Arabidopsis encoding a putative methyltransferase is required for cell adhesion and co-ordinated plant development. Plant J. 2007;50(4):735–50. doi: 10.1111/j.1365-313X.2007.03123.x 17461780

[35] Daher FB, Braybrook SA. How to let go: pectin and plant cell adhesion. Frontiers in plant science. 2015;6:523. doi: 10.3389/fpls.2015.00523 26236321 PMC4500915

[36] Wolf S, Hofte H. Growth Control: A Saga of Cell Walls, ROS, and Peptide Receptors. Plant Cell. 2014;26(5):1848–56. doi: 10.1105/tpc.114.125518 24808052 PMC4079354

[37] Verger S, Chabout S, Gineau E, Mouille G. Cell adhesion in plants is under the control of putative O-fucosyltransferases. Development. 2016;143(14):2536–40. doi: 10.1242/dev.132308 27317803 PMC4958334

[38] Sola K, Gilchrist EJ, Ropartz D, Wang L, Feussner I, Mansfield SD, et al. RUBY, a Putative Galactose Oxidase, Influences Pectin Properties and Promotes Cell-To-Cell Adhesion in the Seed Coat Epidermis of Arabidopsis. Plant Cell. 2019;31(4):809–31. doi: 10.1105/tpc.18.00954 30852555 PMC6501606

[39] Vojtek AB, Hollenberg SM, Cooper JA. Mammalian Ras interacts directly with the serine/threonine kinase Raf. Cell. 1993;74(1):205–14. doi: 10.1016/0092-8674(93)90307-c 8334704

[40] Fields S, Song O. A novel genetic system to detect protein-protein interactions. Nature. 1989;340(6230):245–6. doi: 10.1038/340245a0 2547163

