# Supplementary material for: Golgi ELMO1 binds QUA1, QUA2, GAUT9, and ELMO4 and is required for pectin accumulation in Arabidopsis
Source: PLoS One. 2023 Nov 8;18(11):e0293961. doi: 10.1371/journal.pone.0293961 (PMC10631678; doi:10.1371/journal.pone.0293961)

SI Fig 1. Ruthenium Red stained hypocotyls for the indicated genotype. Scale bar indicates 1 mm.

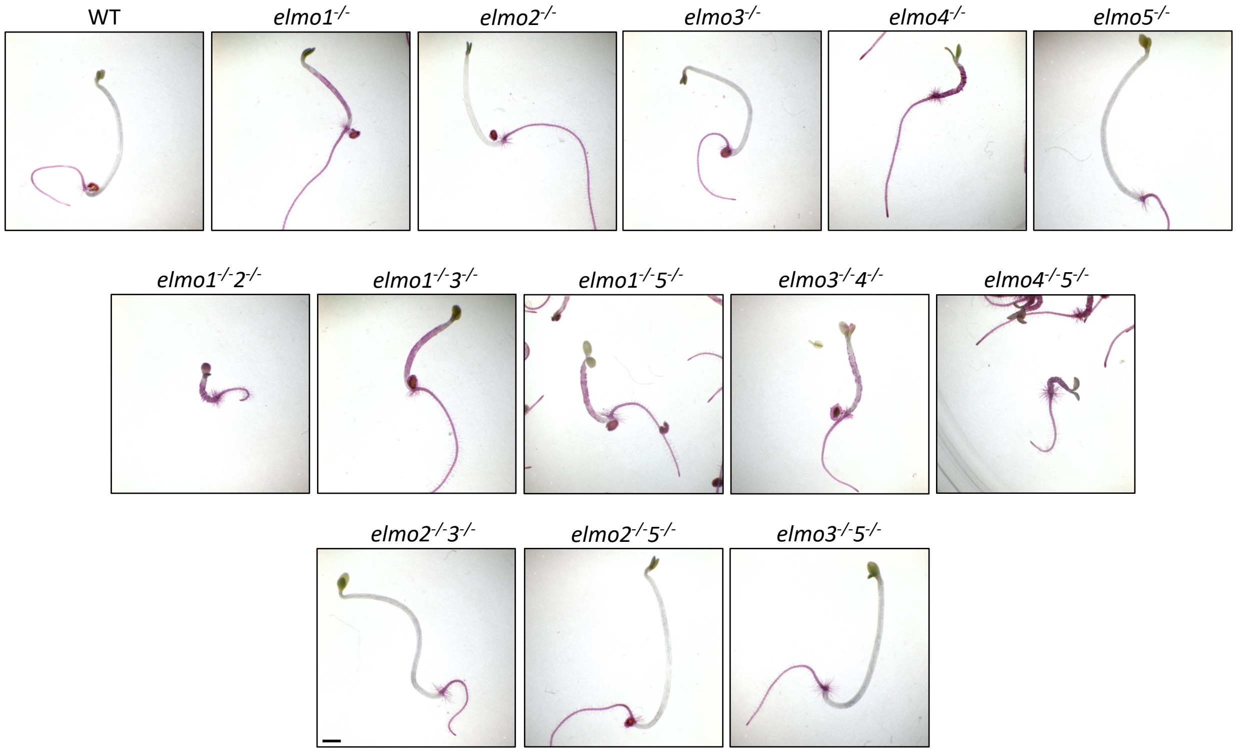

SI Fig 2. elmo mutants have reduced hypocotyl root lengths. 5 day dark grown hypocotyls were imaged using a dissecting microscope and the hypocotyl and roots were measured. Asterisks indicates significant difference from WT (ANNOVA hypocotyl; F (12,337)=91.89 p<0.0001. root; F(12, 312)=20.78 p<0.0001). Bars indicate standard deviation.

SI Fig 2A

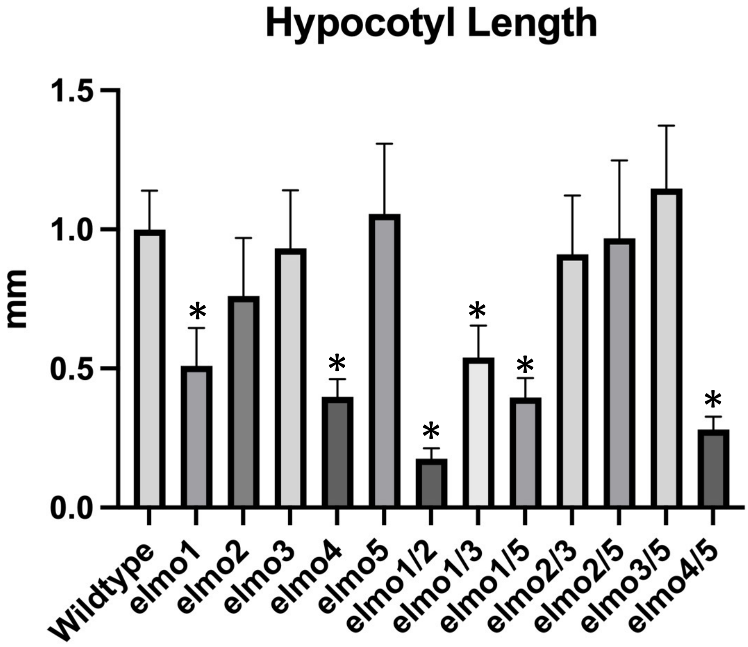

SI Fig 2B

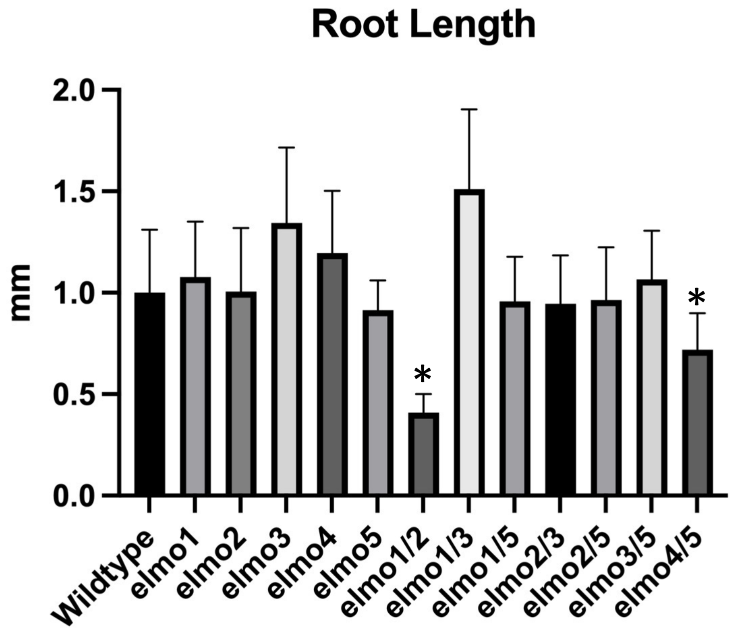

SI Fig 3. Excel files of RNA seq of RNA from elmo1-/-, elmo4-/-, elmo1-/- elmo4-/- and WT dark grown hypocotyls.

SI Fig 4. Excel files of ELMO1-GFP immunoprecipitated from dark grown hypocotyls, and proteins sequenced using SWATH quantitative LC-MS/MS.

Sup Fig 5A

Bait pLEXA	Prey pACT
pLEXA	pACT
ELMO4	pACT
QUA1	pACT
QUA2	pACT
GAUT9	pACT

-Leu Trp Ura

-THULL, +3AT

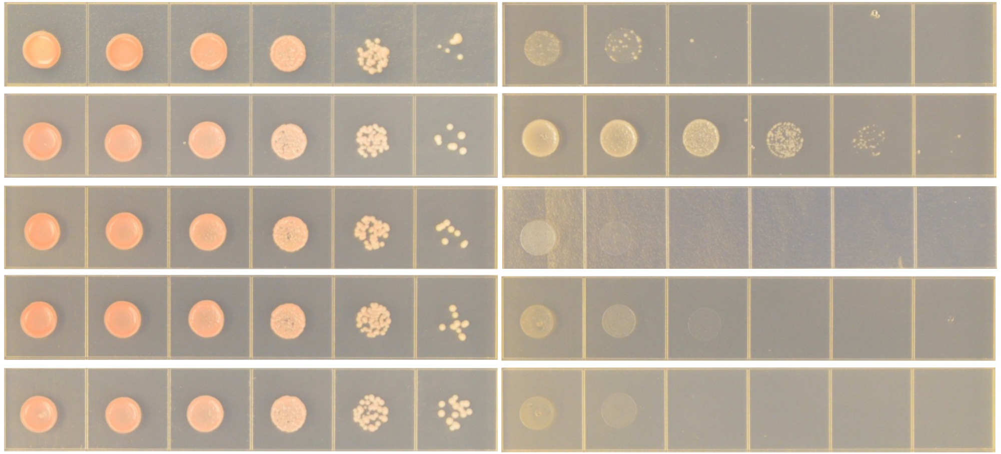

Sup Fig 5B

Bait pLEXA	Prey pACT
pLEXA	pACT
pLEXA	ELMO1
ELMO1	ELMO1
QUA1	ELMO1
QUA2	ELMO1
GAUT9	ELMO1

-Leu Trp Ura

-THULL, +3AT

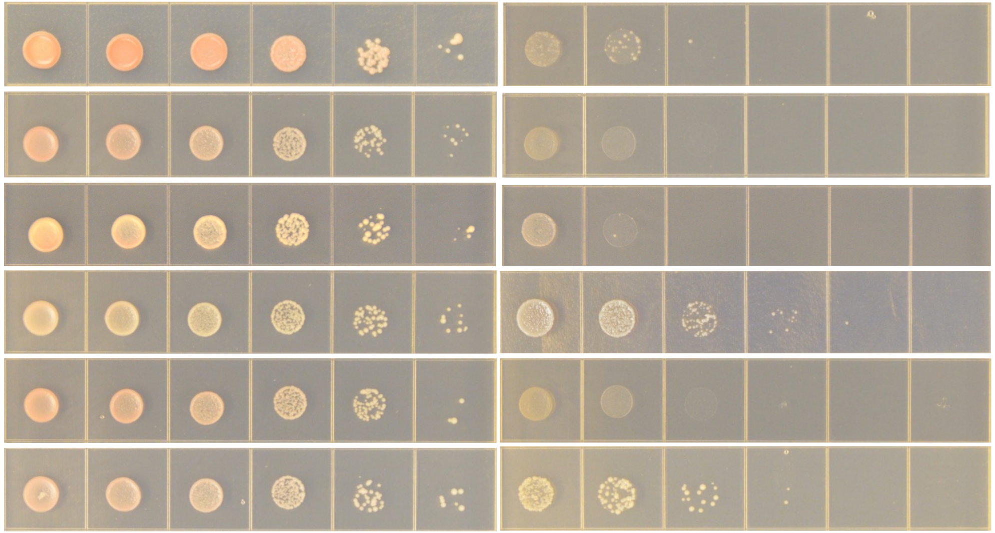

Sup Fig 5C

Bait pLEXA	Prey pACT
pLEXA	pACT
pLEXA	ELMO1
ELMO1	ELMO1
QUA1	ELMO1
QUA2	ELMO1
GAUT9	ELMO1

-Leu Trp Ura | -THULL, +3AT

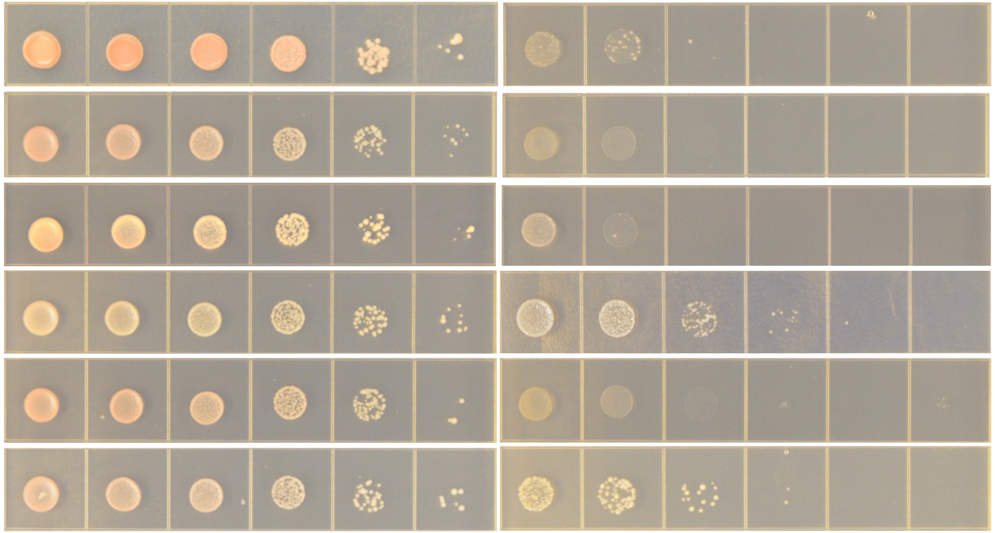

Sup Fig 5D

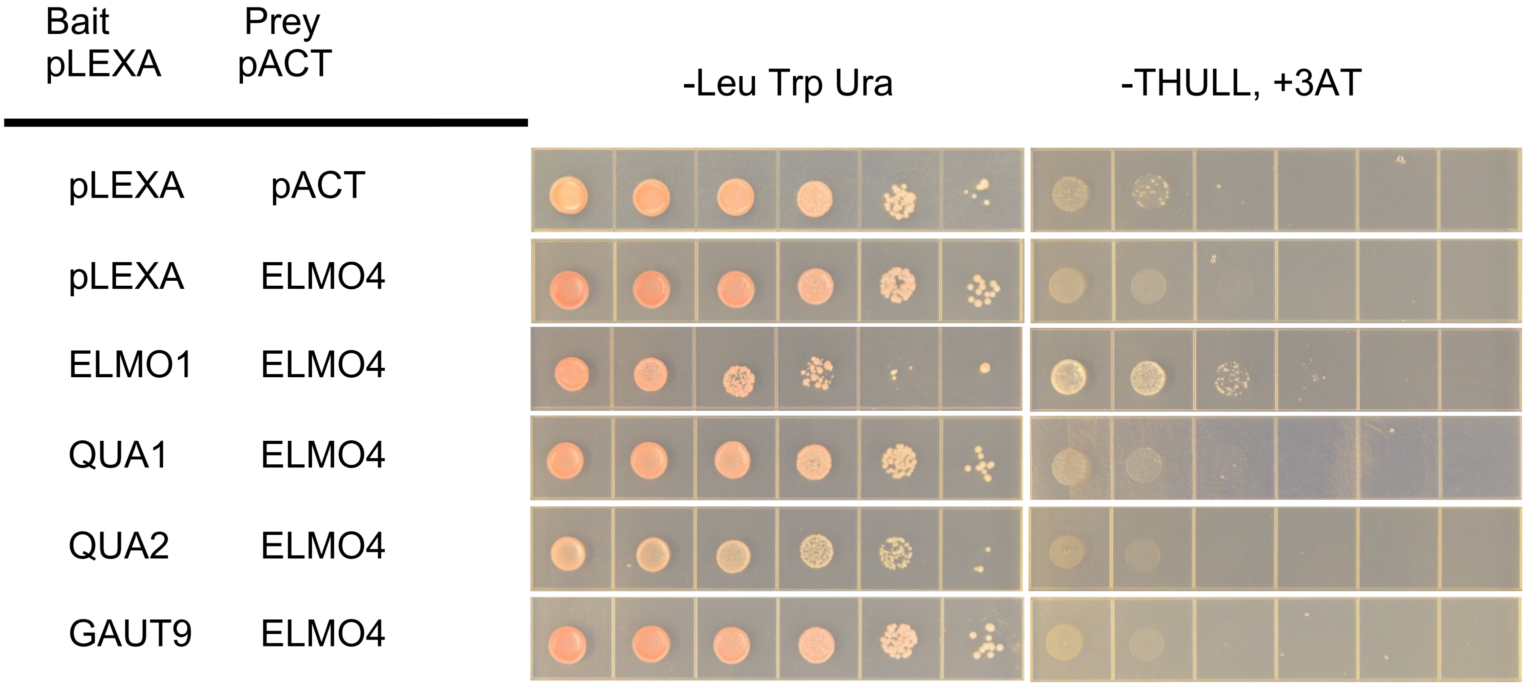
Bait
pLEXA
Prey
pACT
-Leu Trp Ura
-THULL, +3AT
pLEXA pACT
pLEXA ELMO4
ELMO1 ELMO4
QUA1 ELMO4
QUA2 ELMO4
GAUT9 ELMO4

Sup Fig 5E

Bait pLEXA	Prey pACT
pLEXA	pACT
pLEXA	QUA1
QUA1	QUA1
GAUT9	QUA1

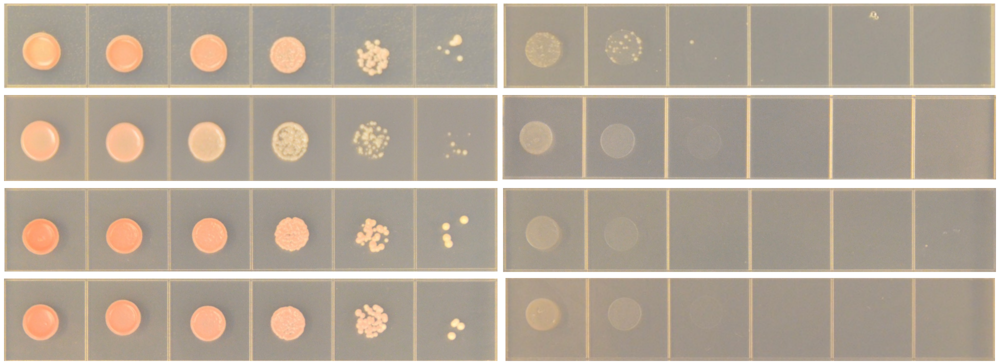

Sup Fig 5F

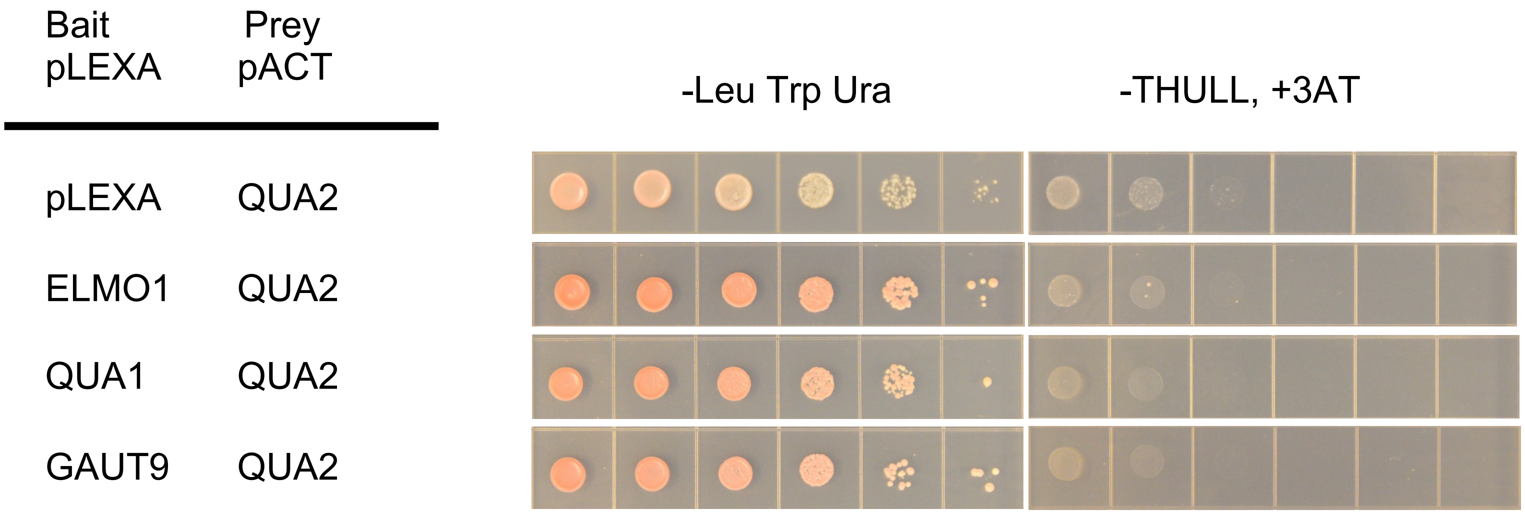
Bait
pLEXA
Prey
pACT
-Leu Trp Ura
-THULL, +3AT
pLEXA QUA2
ELMO1 QUA2
QUA1 QUA2
GAUT9 QUA2

Sup Fig 5G

Bait pLEXA	Prey pACT
pLEXA	GAUT9
QUA1	GAUT9
GAUT9	GAUT9

-Leu Trp Ura

-THULL, +3AT

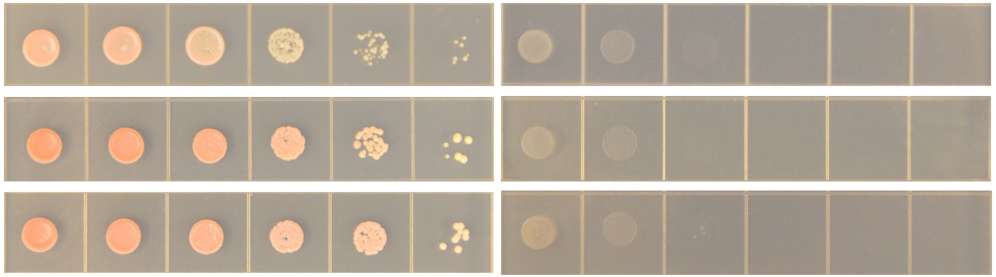

Supplement: S1 File — SI Fig 1. Ruthenium Red stained hypocotyls for the indicated genotype. Scale bar indicates 1 mm. SI Fig 2. elmo mutants have reduced hypocotyl root lengths. 5 day dark grown hypocotyls were imaged using a dissecting microscope and the hypocotyl and roots were measured. Asterisks indicates significant difference from WT (ANOVA hypocotyl; F (12,337) = 91.89 p<0.0001. root; F(12, 312) = 20.78 p<0.0001). ß SI Fig 3 and Fig 4. Zip file containing RNA seq and immunoprecipitation data files. SI Fig 3; Excel files of RNA seq of RNA from elmo1-/-, elmo4-/-, elmo1-/- elmo4-/- and WT dark grown hypocotyls. SI Fig 4; Excel files of ELMO1-GFP immunoprecipitated from dark grown hypocotyls, and proteins sequenced using SWATH quantitative LC-MS/MS. SI Fig 5. Yeast two hybrid assay for the indicated combinations of expressed proteins. (PDF) [file pone.0293961.s001.pdf]
